# Detuned Plasmonic Bragg Grating Sensor Based on a Defect Metal-Insulator-Metal Waveguide

**DOI:** 10.3390/s16060784

**Published:** 2016-05-28

**Authors:** Shinian Qu, Ci Song, Xiushan Xia, Xiuye Liang, Baojie Tang, Zheng-Da Hu, Jicheng Wang

**Affiliations:** 1 School of Science, Jiangsu Provincial Research Center of Light Industrial Optoelectronic Engineering and Technology, Jiangnan University, Wuxi 214122, China; qu.shinian@163.com (S.Q.); Songci77725@163.com (C.S.); xiaxiushan123@163.com (X.X.); liangxiuye121@126.com (X.L.); tangbaojie93@163.com (B.T.); huyuanda1112@jiangnan.edu.cn (Z.-D.H.); 2 Key Laboratory of Semiconductor Materials Science, Institute of Semiconductors, Chinese Academy of Sciences, Beijing 100083, China

**Keywords:** plasmonics, Bragg grating sensor, metal-insulator-metal, finite element method

## Abstract

A nanoscale Bragg grating reflector based on the defect metal-insulator-metal (MIM) waveguide is developed and numerically simulated by using the finite element method (FEM). The MIM-based structure promises a highly tunable broad stop-band in transmission spectra. The narrow transmission window is shown to appear in the previous stop-band by changing the certain geometrical parameters. The central wavelengths can be controlled easily by altering the geographical parameters. The development of surface plasmon polarition (SPP) technology in metallic waveguide structures leads to more possibilities of controlling light at deep sub-wavelengths. Its attractive ability of breaking the diffraction limit contributes to the design of optical sensors.

## 1. Introduction

Surface plasmon polaritions (SPPs) are mixed electromagnetic waves confining at the metal surface, which results from electromagnetic waves coupling to free electron oscillations. There are two specific kinds of plasmonic structures: insulator-metal-insulator (IMI) and metal-insulator-metal (MIM). However, the MIM waveguides are promising for nanoscale applications owing to their attractive feature of light confinement beyond the diffraction limit. Although MIM has more transmission loss, it can be neglected in nano-scale devices [1,2]. In the past few years, several novel photonic devices based on SPPs, such as triangular waveguides [3], absorption switches [4], reflectors [5,6,7], and absorbers [8] have been investigated. Particularly, the MIM Bragg reflectors have a wide range of applications in optical communication fields such as optical filters [9], which have been theoretically proposed and experimentally demonstrated, single-cavity and multi-cavity structures filters [10], and tunable channel drop filters [11].

Due to their unique feature of subwavelength of confinement, optical sensors are another important application of communication that can be controlled by the width [12,13], effective refractive index [14], force, and so on. Some devices based on optical waveguides [15], directional couplers [16], were demonstrated few years ago. In order to measure the change in refractive index within a single sensing spot, several authors have proposed a nanoplasmonic ring hole interferometric sensor [17]. In addition, an ultra-compact loop-stub biosensor and structure of metal nanoslit arrays have been proposed [18,19]. In order to improve the sensing performance straightforwardly, Zhengqi Liu presents a sensor based on the suspended plasmonic crystal [20], a nanostructured X-shaped plasmonic sensor has been designed for investigation phase interrogation [21]. A mechanical sensor has been presented to achieve trapping and releasing of light and high-speed rainbow trapping and releasing [22,23].

In this paper, we present and numerically analyze a Bragg grating based on a MIM structure, mainly studying the dependence of its transmission spectra on the geometrical parameters of the grating. An easily tuned stop-band has appeared in the spectra, and, after introducing a defect in the center of the grating, an open window was obtained in the stop-band transmission spectra. We investigate the sensing characteristics of the graded MIM plasmonic Bragg grating, and the tunability of the proposed structure shows a promising future for various applications.

## 2. Structures and Theoretical Analysis

**Figure 1:**
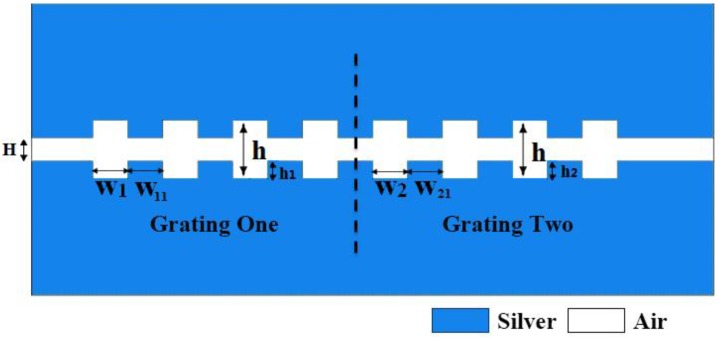
The structure of the MIM plasmonic Bragg grating.

The designed sensor based on MIM structure is shown schematically in Figure 1, where the gap filled with air is inserted to separate silver. We regard the structure as two integrated grating with the same material. The main parameters of the structure are the depth, width of the grating, two widths of the waveguide and the distance between two segments, *h*_1_ (*h*_2_), *w*_1_ (*w*_2_), *h*, *H*, *w*_11_ (*w*_21_) denote them, respectively. Chen has already figured out that as effective *n* = (*w*_1_ + *w*_11_)/*w*_1_ rises, the dispersion curve approaches that of the structured metal wires [24]. In this paper, we assume *w*_1_ = *w*_11_, *w*_2_ = *w*_21_, and *H* stays at the same in all conditions that we will discuss. The light can be coupled into the Bragg grating sensor by nanofiber, and the output light can be detected by microscopy [25]. We use COMSOL (COMSOL Inc., Stockholm, Sweden) to realize our simulations. Input and output are set as port 1 and port 2, respectively, and transverse magnetic (TM) modes are incident from port 1. The calculated area is divided by Yee’s mesh with a size of 2 nm. The FEM with scattering boundary condition is employed to investigate the transmission characteristics of the structure.

**Figure 2:**
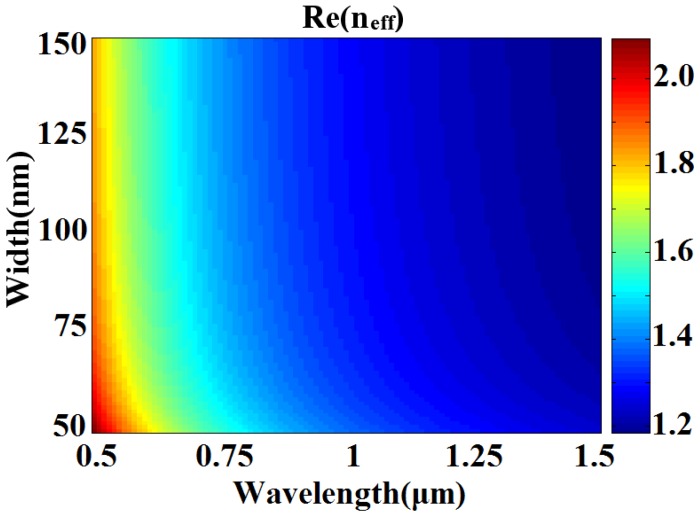
Real part of the effective refractive index *n_eff_* *vs.* the incident wavelength λ and the slit width ω in MIM waveguide.

The dispersion relation of the fundamental plasmonic mode TM_0_ in the MIM waveguide is given by [26]:

(1) $$\tanh\left(\frac{H\sqrt{\beta^{2}-k_{0}^{2}\varepsilon_{i}}}{2}\right)=\frac{-\varepsilon_{i}\sqrt{\beta^{2}-k_{0}^{2}\varepsilon_{m}\left(\omega \right)}}{\varepsilon_{m}\left(\omega \right)\sqrt{\beta^{2}-k_{0}^{2}\varepsilon_{i}}}$$

where ε*_m_* and ε*_i_* are the dielectric constants of the silver and air, respectively. *k*_0_ is the wave vector of light in vacuum. The *n_eff_* = β*/k*_0_ can be calculated by Equation (1). The real part of *n_eff_* as a function of ω and λ is shown in Figure 2. When ω is determined, Re (*n_eff_*) will change little with the increase of the incident wavelength λ.

For its low absorption, silver is chosen in the MIM plasmon whose equivalent permittivity is given by the well-known Drude model [27]:

(2) $$\varepsilon_{m}\left(\omega \right)=\varepsilon_{\infty }-\frac{\omega_{p}^{2}}{\omega \left(\omega +i\gamma \right)}$$

where ε*_∞_* = 3.7 is the dielectric constant at the infinite frequency, γ = 2.73 × 10^13^ Hz is the electron collision frequency, ω*_p_* = 1.38 × 10^16^ Hz is the bulk plasma frequency, and ω stands for the angular frequency of the incident electromagnetic radiation.

For the central wavelength of the stop-band, the well-known Bragg condition formulating the Bragg wavelength is as follows:

(3) w1Re(neff1)+w11Re(neff2)=mλ0/2

where *n_eff_*_1_ and *n_eff_*_2_ are the MIM waveguide mode effective indices of larger and smaller segments of the Bragg grating, *w*_1_ (*w*_2_) and *w*_11_ (*w*_21_) represent the adjacent width of grating in a period, *m* is an integer, and λ_0_ is the Bragg wavelength. From the equation above, we know that the central wavelength of Bragg grating can be easily tuned by controlling the value of *w*_1_ (*w*_2_) and *w*_11_ (*w*_21_) or altering the difference between the effective indices inside the grating. We start our discussion with keeping grating one equal to grating two. The two-dimensional numerical simulations are carried out in the configurations using the FEM.

## 3. Results and Discussion

**Figure 3:**
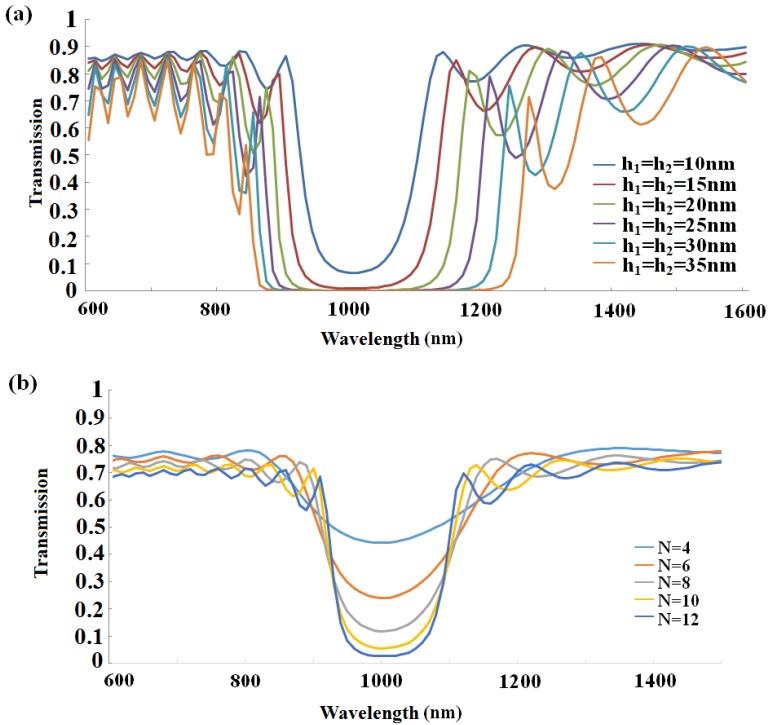
Transmission spectra of the MIM-based sensor as a function of wavelength (**a**) transmission spectra with different air groove grating depth when period number *n* = 8, groove width *w*_1_ = *w*_2_ = 200 nm; (**b**) transmission spectra with different period numbers, with groove depth *h* = 10 nm, groove width *w*_1_ = *w*_2_ = 200 nm.

In order to realize the sensing application of the proposed MIM plasmonic Bragg grating, we optimize the structure parameters by investigating their effects on the transmission spectra. Figure 3a shows the transmission spectra of the MIM-based Bragg grating with different grating groove depth, while *w*_1_ = *w*_2_ = 200 nm, *n* = 8 are fixed. With the depth increasing, the central wavelength displays a red shift varying from 1 μm to 1.05 μm, and the band-gap gets widened dramatically. There are some sidelobes exhibited out of the bandgap, which is probably owing to the light scattering at the abruptly disappearing boundary at the end of the Bragg gratings. It is obvious that, with the increase of the Bragg period number, the dip gets deeper, which is similar to the fiber Bragg grating, but the Bragg wavelength stays almost unchanged, as shown in Figure 3b. However, the total period number of the MIM-based grating is smaller than that of the fiber brag grating (FBG), due to the effective index modulation strength of the MIM grating being much higher than the FBG. Two reasons are listed to decrease the total period number: First, transmission loss will be lower after long-distance metal absorption; second, the more compact structures can be achieved, which is expected for highly integrated circuit structures. Obviously, as the period number goes down, the dip gets shallower and attenuation gets lower. In order to clearly distinguish the Bragg wavelength at transmission dip, we choose *n* = 8 for the following simulation, if not specifically mentioned. In addition, it is important to point out that a great decrease in band-pass transmission efficiency will also be caused by a large period number.

**Figure 4:**
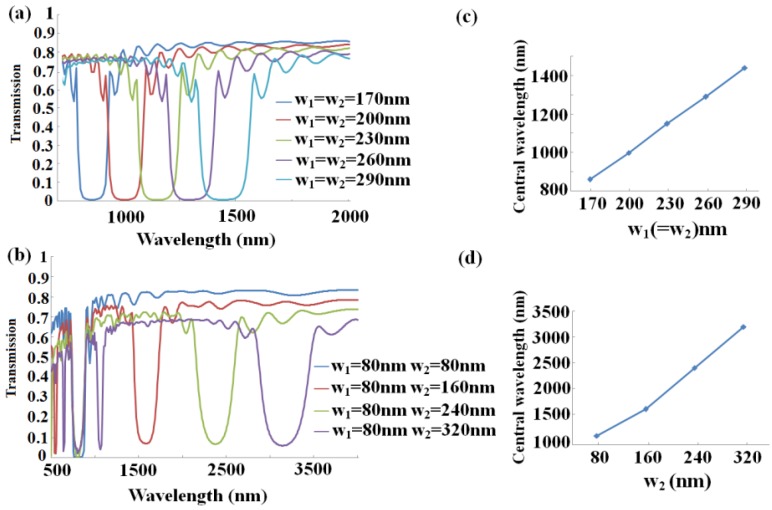
Transmission spectra of the MIM-based sensor as a function of wavelength and the central wavelength as a function of the grating width with *n* = 8, and *h*_1_ = *h*_2_ = 10 nm (**a**) the transmission spectra with grating width *w*_1_ equal to *w*_2_ and increasing at the same time and (**c**) only with *w*_2_ altering and *w*_1_ = 80 nm being fixed; (**b**,**d**) the central wavelength as a function of the grating width corresponding to (**a**) and (**c**), respectively.

As we know, the width modulation can be achieved by altering structure parameters. Thus, we investigate parameter effects on the transmission property. From the Bragg condition, the central wavelength of the stop-band depends on the value of structure parameter *w*_1_ and *w*_2_ with the effective indices remaining unchanged. We choose *n* = 8 for both gratings, *w*_1_ equals to *w*_2_, changing from 170 nm to 290 nm, and increasing 30 nm at a time. As can be seen from Figure 4a, the Bragg wavelength increases with the expansion of the width, which is in accordance with Equation (3). The transmission spectra *vs.* wavelength with different width *w*_2_ have been depicted in Figure 4b, where *w*_1_ is fixed at 80 nm and other parameters stay the same. As the value of *w*_2_ increases from 80 nm to 320 nm, the first band-gaps of four spectra overlap at around 700 nm, but the central wavelength of the second one exhibits a red-shift and the band gap becomes wider, which also agrees well with Equation (3). Obviously, most linear responses in Figure 4c,d clearly depict the central wavelength as a function of the grating width in the two conditions mentioned above.

**Figure 5:**
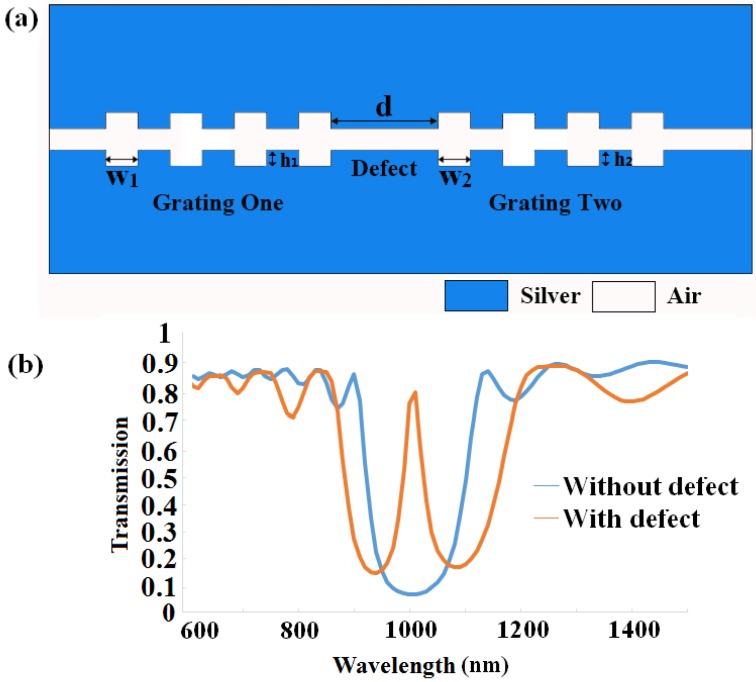
(**a**) The structure of the MIM plasmonic Bragg grating without defect; (**b**) comparison of the Bragg grating and the Bragg grating with a defect *d* = 400 nm.

Then, we insert a nano-cavity in the center of the structure to better understand the characteristics of the MIM-based grating, as been depicted in Figure 5a, where *n* = 6, *w*_1_ = *w*_2_ = 200 nm is used for simulation, with other parameters staying unchanged as at the beginning of our discussion. After introducing a defect into this structure, a narrow transmission peak appears in the center of the band-gap, which is illustrated in the solid orange line in Figure 5b. The solid blue line, depicted as the transmission spectra of the precious structure without defect, is also shown in order to be compared. Figure 5b shows that the wavelength of the new electromagnetic mode is 1 μm, which is exactly the central wavelength of the rejection band without defect when we choose *d* = 400 nm. The *Q* factor of the structure with a defect, *Q* = λ_0_/Δλ, where λ_0_ and Δλ represent the central resonance wavelength and the full width at half-maximum (FWHM) of the defect mode, respectively, describes the ratio of the energy stored in the defect at resonance to the energy escaping from the cavity per cycle of oscillation. According to the theory mentioned above, we can figure out that the *Q* factor for the cavity in Figure 5b is approximately 20.

**Figure 6:**
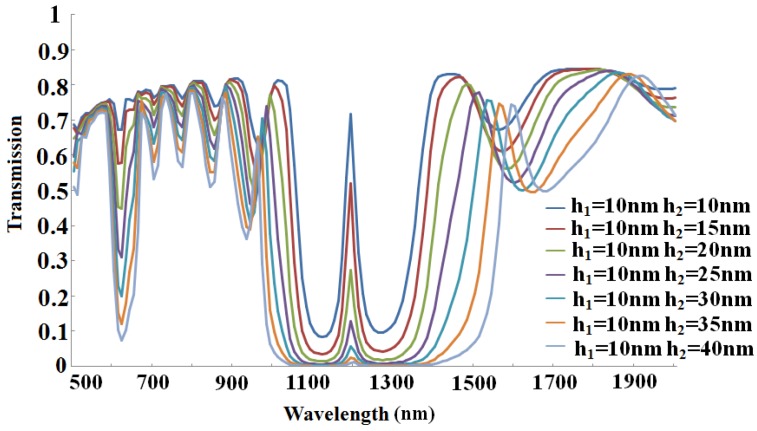
Transmission spectra as a function of wavelength with different heights of later grating with former grating unchanged.

Before starting the discussion about width, we look into graded height of the Bragg grating first. Here, we only change the height of grating two (*h*_2_). In Figure 6, we can see that the transmission maximizes at the same Bragg wavelength, approximately around 1200 nm, but the peak value goes down sharply as grating gets deeper, and when *h*_2_ = 40 nm, three times deeper than *h*_1_, it almost disappears. Meanwhile, the *Q* factor drops quickly, from 30 to 20, which is partly caused by the great energy loss in the transmission process. Therefore, the values we choose that *h*_1_ and *h*_2_ are equal to 10 nm are reasonable choices for better understanding our structure.

**Figure 7:**
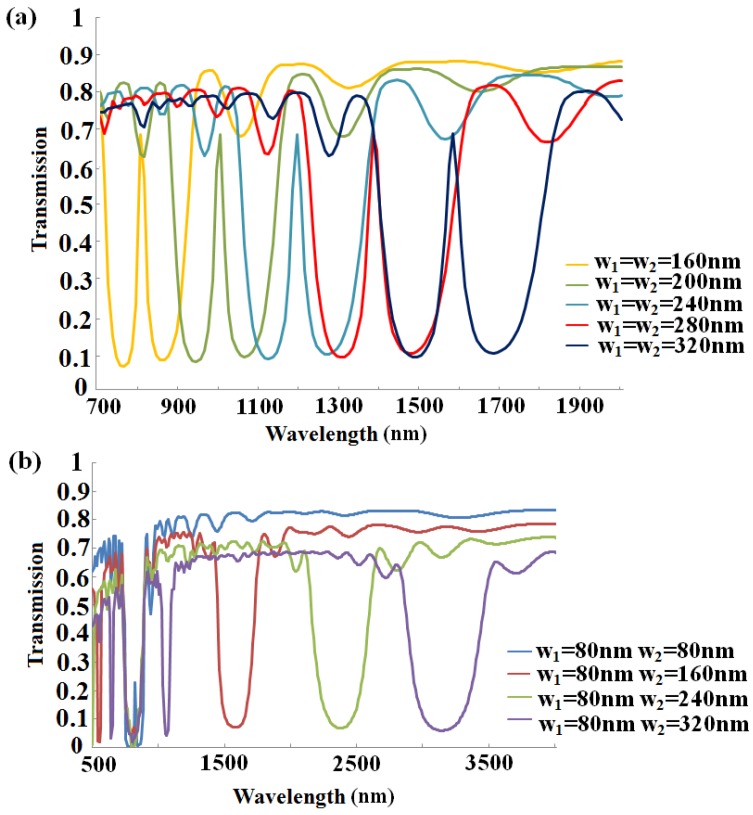
(**a**) The transmission spectra with grating width *w*_1_ and *w*_2_ increase together; (**b**) the transmission spectra with grating width *w*_2_ changing and *w*_1_ = 80 nm being fixed.

Then, the sensing characteristic of the presented structure is investigated by changing the width of the grating. As expected, the transmission window in the stop-band shows a red-shift when we increase the width together, as depicted in Figure 7a. However, in Figure 7b, once one width is fixed and another altered, there will be no peak, which means no wavelength can be sensed.

**Figure 8:**
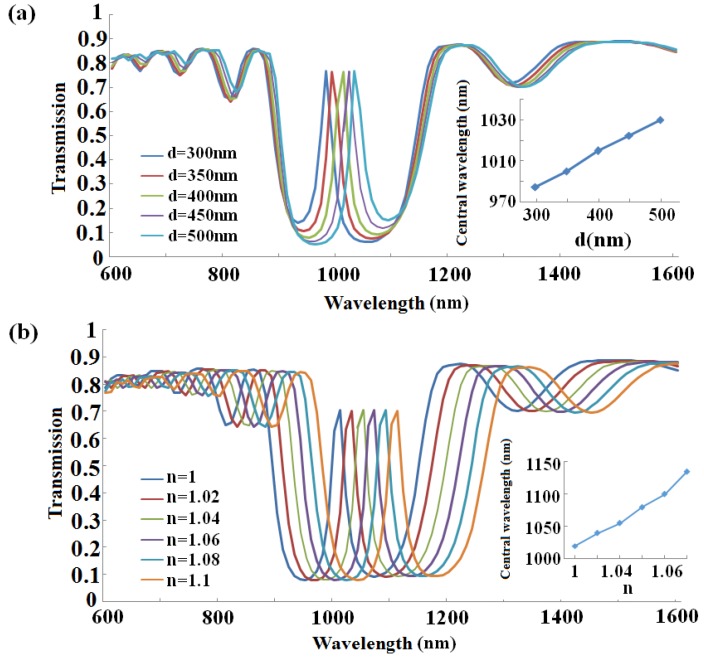
Characterization of the MIM-based Bragg grating with a defect (**a**) transmission spectra as a function of wavelength with different defect width *d*; (**b**) transmission spectra as a function of wavelength with different effective index *n*.

**Figure 9:**
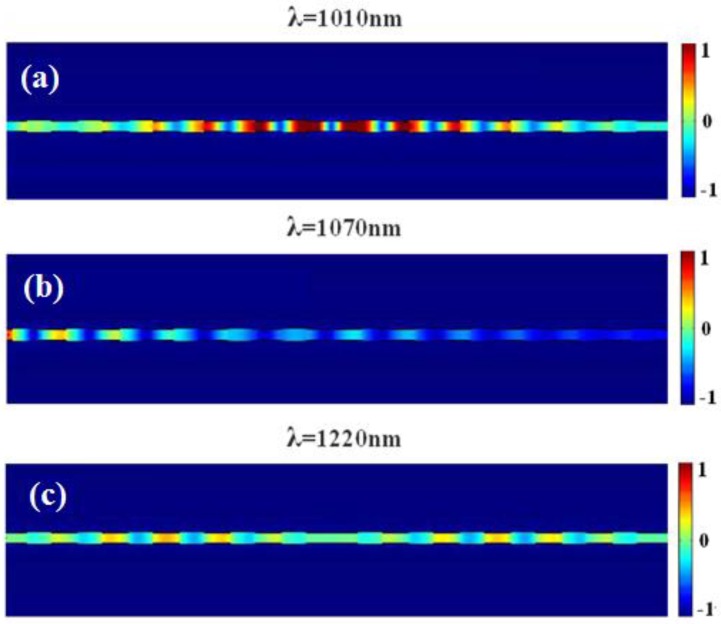
The contour profiles of normalized electric field distributions under different wavelength with defect length *d* = 200 nm (**a**) λ = 1010 nm; (**b**) λ = 1070 nm; (**c**) λ = 1220 nm.

As we can see from Figure 8, the spectra are red-shifted, and the peak of the curve tends towards longer wavelengths with an increase of the defect length or refractive index of the material to be sensed. *Q* factor shows an increasing trend due to λ_0_ keeping unchanged. Importantly, stronger sensitivity of the sensor is obtained. For example, the *Q* factor for the defect cavity is found to be around 24 in Figure 8a with *w*_1_ = *w*_2_ = 200 nm and *h*_1_ = *h*_2_ = 10 nm. It is higher than the value in Figure 5b. Figure 9a exhibits the electric field at peak wavelength λ = 1010 nm. Obviously, the defect leads to the appearance of the resonance mode, whose energy mostly concentrates on the central defect area as expected. The contour profile of the normalized electric field distributions corresponding to transmission dips and passes for the defect length *d* = 200 nm are depicted in Figure 9b,c.

## 4. Conclusions

To sum up, we studied the sensing characteristics of a structure based on a MIM waveguide. We investigated the influence of period and height of two gratings for better structure parameters and also looked into transmission spectra of a proposed structure with different widths. The defect is introduced in the middle of the MIM Bragg grating mentioned above, and the effects of width and graded-altering height on the transmission spectra are discussed, respectively. The other important parameters of the structure, such as the effective index and the length of the defect are investigated in detail. As expected, a majority of the spectra that we looked into are red-shifted in our specific discussion. More importantly, the *Q* factor shows a bit of an increase when we change the parameters, and the characteristics exhibited in the spectra lead to the conclusion that we can choose the wavelength to be sensed by altering structure parameters. In addition, the proposed structures with sensitivity and ultra-narrow light reflection response could be significant for applications of ultra-compact plasmonic devices, such as the active polarization-adjusted multispectral color filtering, displaying and imaging, spectral selective light reflection, and high performance plasmonic sensing in the fields of gas detection, medical diagnostics. All these analyses would provide guidelines to the design of optical filters, sensors and other devices.
